# fBrake, a Method to Simulate the Brake Efficiency of Laden Light Passenger Vehicles in PTIs While Measuring the Braking Forces of Their Unladen Configurations

**DOI:** 10.3390/s24206602

**Published:** 2024-10-13

**Authors:** Víctor Romero-Gómez, José Luis San Román

**Affiliations:** 1 Universidad Carlos III de Madrid, Departamento de Ingeniería Mecánica, Calculation and Transports (MECATRAN), Avenida de la Universidad, 30 (Edificio Sabatini), 28911 Leganés, Spain; jlsanro@ing.uc3m.es; 2 Instituto Seguridad de los Vehículos Automóviles (ISVA-UC3M), Avenida de la Universidad, 30 (Edificio Sabatini), 28911 Leganés, Spain; 3 Purdue University Northwest, Mechanical and Civil Engineering Department, 2200 169th St., Hammond, IN 46323, USA

**Keywords:** vehicle dynamics, braking systems, brake simulation, vehicle safety, European compliance, MATLAB, SOLIDWORKS, CarSim

## Abstract

This study introduces fBrake, a novel simulation method now designed for use in periodic technical inspections of M_1_ and N_1_ vehicle categories, addressing challenges posed by Directive 2014/45/EU. The directive mandates that braking efficiency must be measured relative to the vehicle’s maximum mass, which often results in underperformance during inspections due to vehicles typically being unladen. This discrepancy arises because the maximum braking forces are proportional to the vertical load on the wheels, causing empty vehicles to lock their wheels prematurely compared to laden ones. fBrake simulates the braking forces of unladen vehicles to reflect a laden state by employing an optimal brake-force distribution curve that aligns with the vehicle’s inherent braking behavior, whether through proportioning valves or through electronic brake distribution systems in anti-lock-braking-system-equipped vehicles. Our methodology, previously applied to heavy vehicles, involved extensive experimentation with a roller brake tester, comparing the actual braking performances of dozens of vehicles to those of their simulated counterparts using fBrake. The results demonstrate that fBrake reliably replicates the braking efficiency of laden vehicles, validating its use as an accurate and effective tool for braking system assessments in periodic inspections, irrespective of the vehicle’s load condition during the test. This approach ensures compliance with regulatory requirements while enhancing the reliability and safety of vehicle inspections.

## 1. Introduction

Despite continuous developments, road safety still represents a critical global challenge, with the goal of significantly reducing fatalities and serious injuries on our roads. The concept of “Vision Zero”, [1] first introduced in Sweden in 1997, has gained international recognition, including in the European Union, which has adopted the ambitious target of eliminating all road deaths by 2050, as discussed at the last Transport, Telecommunications and Energy Council on 18 June 2024 [2]. This approach, widely supported by academic research [3,4,5,6], advocates for a transportation system in which no death or serious injury is acceptable. In this context, it is not only necessary to drive the development and implementation of advanced vehicle safety technologies, but also to make sure that during Periodic Technical Inspections, every vehicle remains in proper working order. These two approaches are essential to progressing towards this goal of reducing accidents on roads.

The first approach, developing and implementing advanced safety technologies, plays a big role in achieving the goal of reducing the number of road deaths to zero, and it is linked with automatization. New Automated Driving Systems (ADSs) need to be developed and constantly improved to keep up with this aim, but they face challenges in terms of safety and reliability. This study is mainly focused on safety for Technical Inspection, but a further potential goal is related to how different ADSs could take advantage of the working principles of a braking system applied to vehicle platoon control [4,5,6] and help to improve the controllers currently used in these systems.

Before the publication of Directive 2014/45/EU [7] of the European Parliament and of the Council held on 3 April 2014, which is in force at the time of writing, there were several iterations of the regulations regarding vehicle Periodic Technical Inspections (abbreviated as PTIs in English, and ITVs in Spanish). The European Commission, in its communication entitled, “Towards a European Road Safety Area: policy orientations on road safety 2011–2020” [8], proposed halving the number of road fatalities in the Union by 2020 compared to the initial target set for 2010. To achieve this goal, the Commission established seven strategic objectives, determining actions to achieve safer vehicles, policies to reduce the number of injuries, and measures to improve road user safety.

In this context, Periodic Technical Inspections are part of a regime designed to ensure that vehicles are in good condition from both safety and environmental perspectives during their use. This regime primarily encompasses Periodic Technical Inspections as a vital instrument to ensure that vehicles are roadworthy.

The European Parliament and the Council of the European Union approved Directive 2014/45/EU, concerning periodic roadworthiness tests for motor vehicles and their trailers, on 3 April 2014, repealing Directive 2009/40/EC [9]. This directive incorporates and updates the standards outlined in Recommendation 2010/378/EU [10] of the Commission, dated 5 July 2010, on the assessment of defects detected during technical inspections conducted in accordance with Directive 2009/40/EC [9]. It also includes Directive 2010/48/EU [11], which adapts the earlier Directive 2009/40/EC [9] on technical progress, expanding its scope to include provisions for the establishment of inspection centers, their supervisory bodies, and the appointment of inspectors responsible for conducting technical inspections.

Nationally, until 2017, vehicle technical inspections in Spain were regulated by two separate decrees: Royal Decree 2042/1994 [12], dated 14 October, which regulated vehicle technical inspections, and Royal Decree 224/2008 [13], dated 15 February, concerning general installation and operation standards for vehicle inspection stations. To avoid regulatory dispersion and potential duplications, Royal Decree 920/2017 [14], dated 23 October, repealed the previous decrees, establishing a single national framework regulating vehicle technical inspections.

Based on these developments, and in compliance with Spain’s EU accession treaty obligations, as well as the provisions of Article 23.1 of Directive 2014/45/EU [7] of the European Parliament and of the Council, dated 3 April 2014, it was necessary to enact national provisions adapting Spanish legislation to the directive’s requirements.

Therefore, Royal Decree 920/2017 [14], currently in force in the form of its latest update, on 14 September 2022, aims to transpose Directive 2014/45/EU [7] of the European Parliament and of the Council, dated 3 April 2014, into the Spanish legal framework. This decree harmonizes and consolidates the previous Royal Decrees, 2042/1994 [12] and 224/2008 [13], regulating vehicle technical inspections and general standards for vehicle inspection stations.

Furthermore, Article 8 of Royal Decree 920/2017 [14] mandates that the Inspection Procedure Manual for ITV stations must detail the inspection methods established in its annex I (including braking efficiency calculations) to create a truly harmonized inspection procedure across Spain. The current Inspection Procedure Manual (version 7.7.1, effective from 8 May 2024, and in force at the time of writing) [15] continues to reference braking efficiency calculations for light vehicles with a Maximum Authorized Mass or Gross Vehicle Weight (abbreviated as *MAM* or GVW, in English, and MMA in Spanish) of 3500 kg or less (categories M_1_ and N_1_) based on the Mass in Running Order (abbreviated as MiRO/MRO in English, MOM in Spanish) instead of the *MAM*. However, this must be updated in future revisions of the manual to eliminate discrepancies between European Commission directives and Spanish legislation.

The braking efficiency is defined as follows:

(1) $$E\left(\%\right)=\frac{F_{b}}{MAM\cdot g}\cdot 100$$

where the following apply:

*E*: Braking efficiency in %.
*F_b_*: Braking forces of all wheels (sum of brake tester readings for all wheels in N).
*MAM*: Maximum Authorized Mass in kg.
*g*: Gravitational acceleration (approximately 9.8 m/s^2^).

The values for the minimum allowed efficiencies are those appearing in Table 1:

However, (^1^) exception is not present in the transposed Spanish national legal framework [9], which implements a safety-side approach, where every M_1_ vehicle must achieve an efficiency greater than 50%, regardless of whether it is ABS-equipped and/or whether it was type-approved before 1 October 1991.

On the other hand, it is important to emphasize that the braking force that the vehicle can develop (*F_b_*) is limited by either the mechanical components of the vehicle or by the adherence between the tire and the road (or between the tire and the roller brake tester, in the case of PTI testing) during braking. 

Applying Guillaume Amontons’ first law, the maximum braking force (*F_b.max_*) that the vehicle can develop, in terms of adherence, is directly proportional to the weight to which the tires are subjected (*m*∙*g*):

(2) $$\Sigma F_{b.max}=\mu \cdot m\cdot g$$

where the following apply:

Σ*F_b.max_*: Sum of the maximum individual achievable braking forces in N.
*μ*: Adherence coefficient between the tire and the homologous surface.
*m*: Mass of the vehicle in kg.
*g*: Gravitational acceleration (approximately 9.8 m/s^2^).

In practice, this results in an important limitation for vehicles whose gross weight or Maximum Authorized Mass is quite different from their Mass in Running Order. Using a practical example, if a vehicle has a *MAM* of 3500 kg and an MRO of 1750 kg, considering an adherence coefficient between the tire and the roller of 0.9, the maximum braking force, regardless of the condition of the braking system, will be limited to

(3) $$E_{max}\left(\%\right)=\frac{\Sigma F_{b.max}}{MAM\cdot g}\cdot 100=\frac{\mu \cdot m\cdot g}{MAM\cdot g}\cdot 100=\frac{0.9\cdot 1750}{3500}\cdot 100=45\%$$

This is one of the reasons why the fBrake team initially planned a version of software for heavy vehicles as an urgent necessity for Vehicle Testing Facilities [16,17], since the difference between the MRO and *MAM* masses is bigger for these vehicles. The other reason is related to regulations. Heavy vehicles needed to be tested following the standards given by ISO 21069 [18,19] or by equivalent methods, after Directive 2010/48/EU came into force, allowing comparable methods like fBrake to be considered. This fBrake version for heavy vehicles is currently marketed and in use in many PTI facilities in Spain, providing successful results, especially for vehicles that are not easy to inspect when laden, such as buses, or vehicles that involve risk when loaded for inspection, like trucks that deliver dangerous goods. Additionally, fBrake is a simulation that does not require the manual measurement of hydraulic or pneumatic pressures directly from the vehicle, so PTI operators are safer at all times.

Other approaches to check the correct operation of the braking system in PTIs are being developed in more EU countries. As an example, a PTI adapter is used in Germany to detect reference values from the On-Board Diagnostic (OBD) port while performing braking efficiency tests based on the aforementioned reference values. If the PTI adapter is used as a deceleration measurement device, the efficiency of the braking system can also be assessed during a test drive [20]. However, this method is not able to extrapolate efficiency to another loading state, on which fBrake focuses. The PTI adapter can be used for the diagnostics of the hydraulic pressure modulators of ABS/ESP systems in standing conditions [21].

Currently, considering the way in which Directive 2014/45/EU is phrased, simulations or additional methods to evaluate the braking efficiency for M_1_ and N_1_ vehicles are not contemplated. However, it is feasible that a future version of the directive will include this option, so in preparation, this fBrake for M_1_ and N_1_ vehicles was developed. Normally, light-vehicle owners attend the PTI alone, so those vehicles’ masses are closer to the MRO than the *MAM*. Calculating the braking efficiency with respect to the *MAM* with vehicles evaluated in a driver-only configuration would lead to quite a high rejection rate due to the explanation covered by (3), or to a time-consuming and uncomfortable inspection for the vehicle owner, since the operators would need to load the vehicle with extra weight until the *MAM* condition was met. A simulation method like fBrake will be key for the convenience of both PTI facilities and their users.

## 2. Materials and Methods

The fundamentals of fBrake method rely on the ideal brake-force distribution curves, because these represent the behavior every braking system tries to replicate, either with proportioning valves or using Electronic Brake-Force Distribution (EBD). The former were used to prevent rear-wheel locking by limiting rear brake force. However, this led to inefficiencies because proportioning valves try to adapt a curve using two slopes, and this approximation is too conservative for the rear axle. EBD, on the other hand, adjusts brake force dynamically to ensure maximum rear brake usage without wheel locking, improving overall braking efficiency and stability.

**Figure 1:**
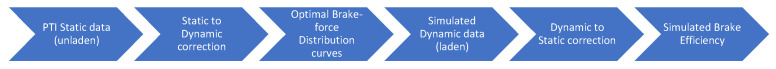
fBrake simulation workflow.

As shown in Figure 1, fBrake models use the braking force measurements obtained at the PTI facility in an unladen state (closer to MRO, closer to *MAM*, or anything in between), which are braking forces developed in static conditions (without mass transfer), to finally obtain the braking forces that the same vehicle would achieve under static load (without mass transfer), but laden. For this purpose, the optimum brake-force distribution curves are used with the braking force data measured at the PTI facility corrected to estimate the braking forces that would have been obtained from a dynamic test (with mass transfer). This is because optimum brake-force distribution curves, constant deceleration lines and adhesion lines have the mass transfer effect implied.

To calculate the optimal brake-force distribution curve for the unladen condition, there is a need to correct the static forces measured during PTI inspection into a dynamic equivalent, considering load transfer. This extrapolation can be achieved regardless of vehicle’s transmission type (FWD, RWD, AWD, etc.) because tests are performed in neutral.

The forces involved in a dynamic longitudinal braking test are the inertial force, weight, frictional force, braking force, aerodynamic drag and rolling resistance. However, the latter two are not considered on the diagram due to the following:

1. The aerodynamic drag does not have any effect on our study because the weight transfer transformation is given as a method to allow us to use the optimal brake-force distribution curve, but the car is not moving, and the effect of the aerodynamic drag is negligible.
2. The frictional force of the transmission does not affect the result (vehicle in neutral, clutch is uncoupled), and frictional force produced by mechanical components up to the clutch is calculated by the roller brake tester before each test, so this force has already been subtracted from the braking forces measured by the system.

The dimensions involved in a dynamic longitudinal braking test are the longitudinal distances between the Center of Gravity (CG) and the front and rear axles, as well as vertical position of the vehicle’s CG (*CG_h_*).

**Figure 2:**
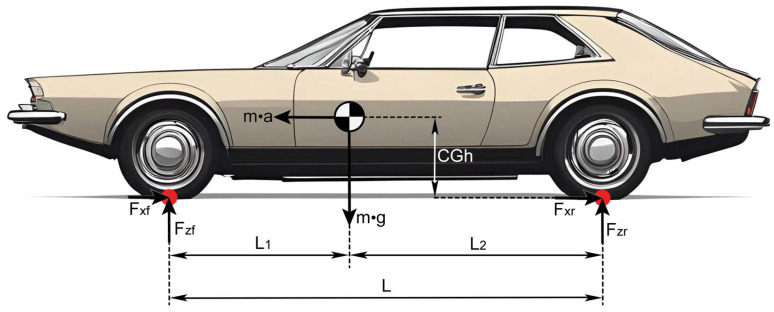
Diagram of forces involved in a dynamic longitudinal test.

The nomenclature of the variables shown in the Figure 2 is as follows:

*F_xf_*: Front longitudinal force between tires and ground. This is the front braking force.
*F_zf_*: Front normal force between tires and ground. This is the front weight.
*F_xr_*: Rear longitudinal force between tires and ground. This is the rear braking force.
*F_zr_*: Rear normal force between tires and ground. This is the rear weight.
*m·g*: Total weight of the car.
*m·a*: Inertial force.

At the same time, the braking force and the friction force between the wheels and the roller are directly related to each other. The maximum braking force will be limited by either the available adherence between the wheel and the roller/mating surface or by the braking system itself.

For the forces and dimensions shown in Figure 2, it is possible to obtain the ideal brake-force curve for the initial loading state by analyzing the force equilibrium and the moment equilibrium in the contact areas between the front tires and the ground, as well as that between the rear tires and the ground.

Moments:

(4) $$\Sigma M_{f}=0={CG}_{h}\cdot m\cdot a+F_{zr}\cdot L-m\cdot g\cdot L_{1}$$

(5) $$F_{zr}=\frac{m\cdot g\cdot L_{1}-{CG}_{h}\cdot m\cdot a}{L}$$

(6) $$F_{zr}=\frac{m\cdot g\cdot L_{1}}{L}-\frac{{CG}_{h}\cdot m\cdot a}{L}$$

(7) $$\Sigma M_{r}=0={CG}_{h}\cdot m\cdot a-F_{zf}\cdot L+m\cdot g\cdot L_{2}$$

(8) $$F_{zf}=\frac{m\cdot g\cdot L_{2}+{CG}_{h}\cdot m\cdot a}{L}$$

(9) $$F_{zf}=\frac{m\cdot g\cdot L_{2}}{L}+\frac{{CG}_{h}\cdot m\cdot a}{L}$$

Forces:

- Longitudinal equilibrium:

(10) $$F_{b.max}=F_{xf}+F_{xr}=m\cdot a$$

- Guillaume Amontons’ first law:

(11) $$F_{b.max}=F_{xf}+F_{xr}=\mu \cdot m\cdot g$$

- Relationship between *F_x_* and *F_z_*:

(12) $$F_{xf}=\mu \cdot F_{zf}$$

Combining (10) and (11), as well as (6) and (9) with (12):

(13) $$F_{xr}=\frac{\mu \cdot m\cdot g\cdot L_{1}}{L}-\frac{{CG}_{h}\cdot \mu^{2}\cdot m\cdot g}{L}$$

(14) $$F_{xf}=\frac{\mu \cdot m\cdot g\cdot L_{2}}{L}+\frac{{CG}_{h}\cdot \mu^{2}\cdot m\cdot g}{L}$$

Thus:

(15) $$F_{xr}=\frac{\mu \cdot m\cdot g}{L}\cdot \left(L_{1}-{CG}_{h}\cdot \mu \right)$$

(16) $$F_{xf}=\frac{\mu \cdot m\cdot g}{L}\cdot \left(L_{2}+{CG}_{h}\cdot \mu \right)$$

Dividing (15) and (16):

(17) $$\frac{F_{xf}}{F_{xr}}=\frac{L_{2}+{CG}_{h}\cdot \mu }{L_{1}-{CG}_{h}\cdot \mu }$$

Solving for *μ*:

(18) $$\mu =\frac{F_{xf}\cdot L_{1}-F_{xr}\cdot L_{2}}{{CG}_{h}\cdot (F_{xf}+F_{xr})}$$

Substituting (18) into (15), the ideal brake-force distribution curve is achieved:

(19) $${\left(F_{xf}+F_{xr}\right)}^{2}+\frac{m\cdot g}{{CG}_{h}}\cdot \left(F_{xr}\cdot L_{2}-F_{xf}\cdot L_{1}\right)=0$$

**Figure 3:**
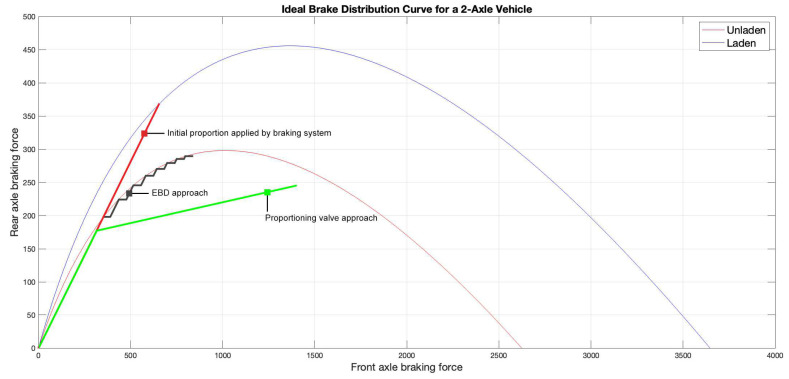
Ideal brake-force distribution curves of a 2-axle vehicle. Units for both axes: daN.

As explained earlier in this section, vehicles with EBD tend to replicate the ideal brake-force distribution curve [22]. In Figure 3, there is an example of two ideal curves, one for a given unladen vehicle (in red) and the other for the same vehicle, but in a laden condition (in blue). The vertical axis shows the rear braking force and the horizontal axis shows the front one, both in daN. The sawtooth pattern represented in black is the result of a cyclic pattern created by the EBD, which keeps applying the initial brake proportion between the front and rear axles until a microslip is detected. At that instant, the rear ABS valves limit the hydraulic pressure, while front ones keep raising it. When rear ABS sensors no longer detect slippage, the braking system applies the pressure with the initial proportion and the cycle stars again. 

It is worth mentioning that braking coordinates must always be below the curve. A coordinate above the curve would imply a lock on the rear axle, which would lead to directional instabilities, involving dangerous situations on the road.

The variables necessary to represent the ideal distribution are the front and rear weights of the car (necessary to obtain *m*, *L*_1_ and *L*_2_), as well as the CG height. A simple axle weighting at the beginning of the inspection (an ordinary task for any PTI station) solves the acquisition of former variables. However, the latter variable (*CG_h_*) is not easy to obtain at all, and a special facility is required to measure it. For the ideal distribution curve for laden condition, the amount of longitudinal displacement that the center of gravity changes backward must be estimated. Side blueprints of dozens of vehicles from each segment were analyzed, along with the positions of the occupants and payloads, to make this estimator.

Regarding *CG_h_*, S-E-A Limited, specifically its Vehicle Testing and Safety division, located in Columbus, OH, USA, features a Center of Gravity and Inertia Testing facility, called Vehicle Inertia Measurement Facility (VIMF). This VIMF is the premier, state-of-the-art system for measuring mass, center of gravity (CG) and inertia properties for a broad range of vehicle types, and it is used by rollover resistance program in United States National Highway Traffic Safety Administration (NHTSA)’s New Car Assessment Program (NCAP), as well as by many independent vehicle manufacturers, to obtain CG data and inertial data for the company’s new models. Using the data found in their latest report regarding CG location measurements [23], the relation between *CG_h_* and wheelbase, total vehicle height and track width for 4 different vehicle segments were evaluated to select the most appropriate correlation as the base starting point for fBrake M_1_ and N_1_ algorithm.

**Figure 4:**
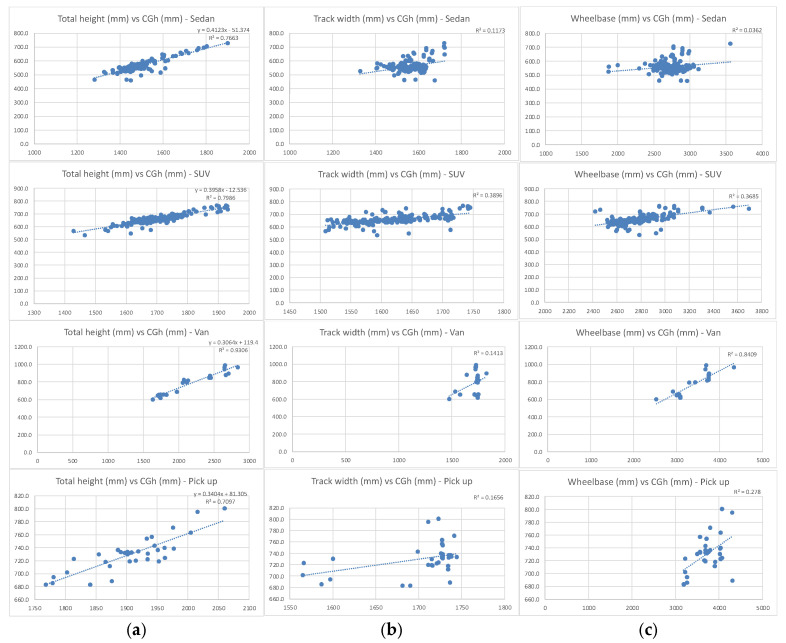
(**a**) Correlation between vehicle total height and *CG_h_*; (**b**) Correlation between vehicle track width and *CG_h_*; (**c**) Correlation between vehicle wheelbase and *CG_h_*.

For every single M_1_ and N_1_ vehicle segment tested (sedan, SUV, van and pick-up truck), all of which were under the light-vehicle definition, the maximum correlation coefficient was achieved when comparing vehicle’s total height vs. *CG_h_* (Figure 4).

A comparison was performed between the following scenarios to prove the effectiveness of fBrake simulation for M_1_ and N_1_ vehicles:

1. Vehicle testing under close-to-laden conditions at PTI facilities.
2. Vehicle testing under unladen conditions at PTI facilities + fBrake simulation.
3. Dynamic brake test on track.
4. CarSim v.8 simulation using inertial data provided by S-E-A Limited.

The comparison between the first scenario and the second was achieved using the data for dozens of vehicles brought by volunteers during 2022 and 2023 to the brake testing facilities located at Universidad Carlos III de Madrid and other collaborating PTI facilities, like ITV Paracuellos del Jarama and ITV Seseña. The comparison between all four scenarios was achieved using one sample vehicle, since for scenario 4, the inertial data for each specific vehicle were needed. After a visit to S-E-A Limited’s facilities in Columbus, OH, the vehicle dynamics team was selfless enough to generously provide us with the inertia data for one vehicle, which we chose to use from a list. Due to its availability in Spain for use as the test vehicle, the 2015 Nissan Juke FWD was selected.

### 2.1. CG_h_ Simulation

**Figure 5:**
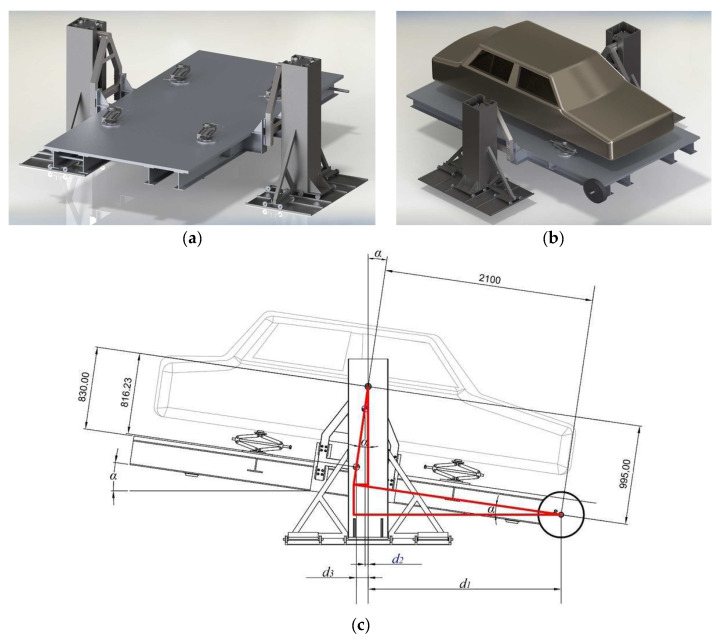
*CG_h_* and pitch inertia measuring facility modeled in SOLIDWORKS. (**a**) Modeled facility without a dummy vehicle on it; (**b**) Modeled facility with a dummy vehicle on it; (**c**) Side blueprint of the assembly.

For instructional purposes and to evaluate the ability of these kinds of facilities to obtain the inertial data of different vehicles, a complete inertial facility was modeled in SOLIDWORKS v.2023 and simulated a realistic measurement of a dummy vehicle *CG_h_* and pitch moment of inertia (Figure 5).

Moment equilibrium equation:

(20) $$m_{1}\cdot \left(2100\cdot cos\alpha -995\cdot sin\alpha \right)=m_{2}\cdot 816.23\cdot sin\alpha +m_{3}\cdot d_{{CG}_{3}\to pin}\cdot sin\alpha$$

where the following apply:

*m*_3_ = vehicle mass.
*m*_2_ = platform mass.
*m*_1_ = calibrated weight.

(21) $${CG}_{h_{vehicle}}=830-\frac{m_{1}\cdot \left(2100\cdot cos\alpha -995\cdot sin\alpha \right)-m_{2}\cdot 816.23\cdot sin\alpha }{sin\alpha \cdot m_{3}}$$

### 2.2. Pitch Moment of Inertia Simulation

Since the platform is mounted as a pendulum on the horizontal transverse axis (pitch), it is easy to calculate the moment of inertia passing through this axis. Moreover, this is also the most interesting angle for this research on braking in longitudinal dynamics. Having balanced the swing with the vehicle mounted and in equilibrium position with the platform completely horizontal, it is easy to exert a force on one of the ends of the platform and let it oscillate, usually until reaching 3 degrees of amplitude. Once the CG height of the vehicle–platform assembly and the period of oscillation are known, it is possible to obtain the moment of inertia about this axis using the following method:

Based on Newton’s Second Law applied to rotation:

(22) $$\sum M_{o}=I_{o}\cdot \ddot{\theta }$$

Equilibrium of moments at the pivot *O*, the only existing force being the one created by the mass of the vehicle–platform assembly applied at the center of gravity of the vehicle–platform assembly, whose position vector from the swing pivot is *r_G_*:

(23) $$\sum M_{0}=-mg\left(r_{G}\cdot \sin\theta \right)-I_{o}\cdot \ddot{\theta }=0$$

By oscillating at a small angle (less than 3 degrees), it is possible to approximate sin *θ* to *θ* and obtain the following second-order homogeneous linear differential equation:

(24) $$\ddot{\theta }+\frac{mgr_{G}}{I_{o}}\theta =0$$

The differential equation is easily solved using the constant coefficient method for second-order homogeneous differential equations.

(25) $$\theta \left(t\right)=\theta_{0}\cos\sqrt{\frac{mgr_{G}}{I_{o}}}t$$

The oscillation frequency and period can be extracted from Equation (25):

(26) $$f=\sqrt{\frac{mgr_{G}}{I_{o}}}\;\;T=2\pi \sqrt{\frac{I_{o}}{mgr_{G}}}$$

Therefore, it follows that the pitch moment of inertia passing through the pivot of the swing is

(27) $$I_{0}=mgr_{G}{\left(\frac{T}{2\pi }\right)}^{2}$$

where the following apply:

*I*_0_: Moment of inertia in the swing pivot (kg·m^2^).
*m*: Mass of the vehicle–platform swing assembly (kg).
*g*: Acceleration of gravity (m/s^2^).
*r_G_*: Distance from the center of gravity of the vehicle–platform swing assembly to the swing pivot (m).
*T*: Period of oscillation (s).

## 3. Results

### 3.1. Scenario 1: Vehicle Testing under Close-to-Laden Conditions at PTI Facilities

For this scenario, different vehicles were tested under laden conditions, or as close as possible to them. Each test consisted of five different runs, which were converted into 10,000 samples using bootstrapping, following the UNE 26110 standard [24], to validate the equivalence of braking efficiency testing methods in relation to the methods defined in ISO 21069. This section emphasizes the 2015 Nissan Juke test as the common thread in the four scenarios. The results of the five brake tests performed in these conditions are shown in Table 2.

### 3.2. Scenario 2: Vehicle Testing under Unladen Conditions at PTI Facilities + fBrake Simulation

For this scenario, five different runs per test were achieved (Table 3), which were later replicated using a custom MATLAB v.2023b code for the bootstrapping.

**Figure 6:**
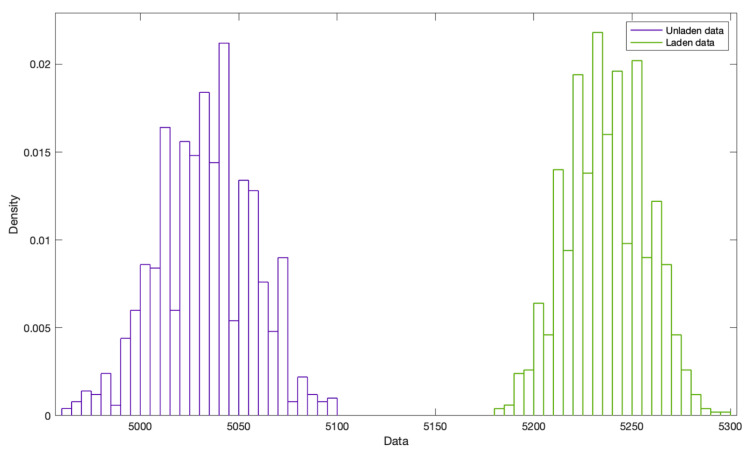
Probability distribution representation of the bootstrapped data for both unladen and close-to-laden tests (X-axis data in N).

The bootstrapping for both scenarios is shown in Figure 6.

Average *F_xf_* for Scenario 1 after bootstrapping: 5236 N.

Average *F_xf_* for Scenario 2 after bootstrapping: 5033 N.

**Figure 7:**
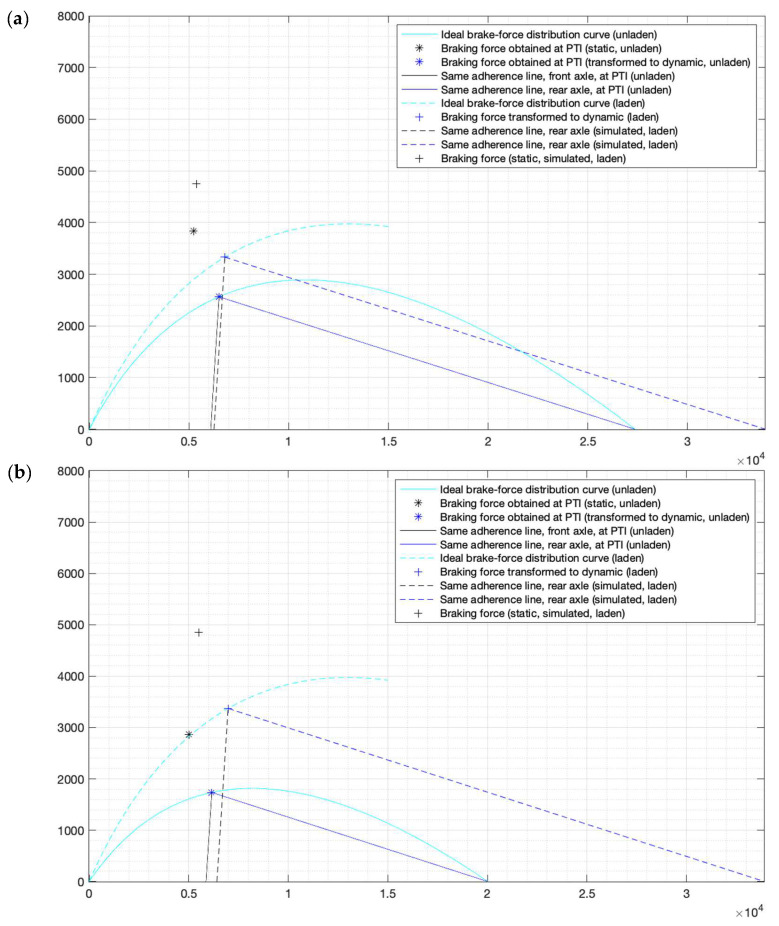
(**a**) fBrake simulation results for Scenario 1; (**b**) fBrake simulation results for Scenario 2. X-axis shows *F_xf_* (N). Y-axis shows *F_xr_* (N).

The testing vehicle used had the specifications shown in Table 4. The ideal brake-force distribution curves for both Scenario 1 and 2 are shown in Figure 7.

The brake efficiency referred to the *MAM* between the two different loading conditions showed a difference of 2.2%, which can be considered quite precise due to the lack of repeatability that is usually related to roller brake testers. Similar results were achieved with the rest of the vehicles brought by the volunteers between 2022 and 2023, validating the working principle of fBrake for N_1_ and M_1_.

It can be observed that attending a PTI with a driver-only configuration (Scenario 2) leads to a misleading failure, resulting in the rejection of the vehicle, if the brake efficiency is directly calculated with PTI-obtained *F_x_* and *MAM* (45.52% < 50%), whereas the fBrake-simulated brake efficiency achieves a value above the required level.

### 3.3. Scenario 3: Dynamic Brake Test on Track

When it comes to justifying the braking Regulatory Act (RA) when necessary, the Department of Mechanical Engineering of Universidad Carlos III de Madrid operates the ISVA-LABITV laboratory in Leganés, Madrid, which is accredited to perform dynamic braking tests, and features a decelerometer manufactured by MAHA, model VZM300, which is capable of determining the average total deceleration according to Directive 71/320/EEC [25], which came into force on 30 July 1971. This Directive was updated through amendments and was finally repealed by Regulation (EC) No. 661/2009 [26] on 31 October 2014, and, subsequently, by Regulation (EU) 2019/2144 [27], which is currently in force with its consolidated version as of 7 July 2024 and requires the use of Regulation No. 13-H [16] to justify the braking RA in passenger cars.

R13-H computes the Average Deceleration applicable on a vehicle with a date of first registration after 31 October 2014, as follows [28]:

(28) $${deceleration}_{average,R13-H}=\frac{{v_{b}}^{2}-{v_{e}}^{2}}{25.92\cdot (s_{e}-s_{b})}$$

However, instead of the decelerometer, to compare the static data and simulations with a dynamic brake test, the vehicle was equipped with a low-cost data acquisition system consisting of a smartphone manufactured by Apple, model iPhone XS Max running the MATLAB Mobile app, and the acceleration data (from an accelerometer sensor), magnetic field data (from a magnetometer), orientation and angular velocity (from a gyroscope), and position (from GPS, GLONASS, Galileo and QZSS receivers) were logged. The dynamic test was performed in Paracuellos del Jarama, Madrid.

For multiple reasons, this research was performed with a low-cost acquisition system. A few of these reasons are as follows:

- The succession of digital acceleration data instead of average acceleration and graph on paper.
- For the calculation of the average acceleration using the raw data, it was possible to choose the desired formula depending on the Directive, Regulation, or other method without depending on the firmware of the device that calculated the acceleration, according to Directive 71/320/EEC.
- Ease of data acquisition was ensured by using the mobile application “MATLAB Mobile” linked to the MathWorks account, creating a *.m file that was uploaded to the MathWorks Cloud automatically after each test.
- The sampling frequencies of all the sensors were up to 100 Hz and manually selectable.
- To test whether such a low-cost sensor can be useful in testing.

**Figure 8:**
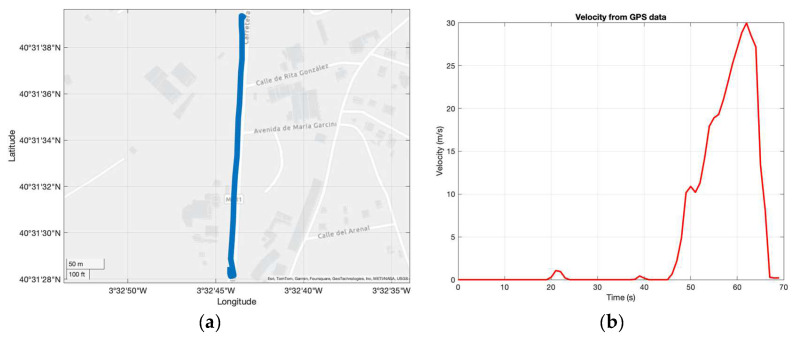
(**a**) GPS data logged by MATLAB Mobile and represented on the map. (**b**) GPS speed.

The phone needed to be attached as closely as possible to the vehicle’s CG. All these logs could be achieved with a sample rate of 100 Hz, which is fast enough for this application, except for position data, which could be logged at 1 Hz, making it unusable in terms of calculating results, although it was helpful in representing the test route on the map (Figure 8).

**Figure 9:**
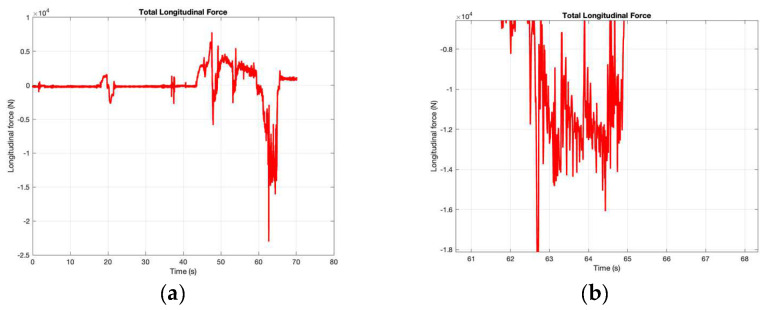
(**a**) Total longitudinal force (N) vs. time (s); (**b**) Closeup view of the highest longitudinal forces.

The most useful graph to plot is that of the longitudinal force vs. time (Figure 9). These data were obtained with the accelerometer sensor, the timecode and the mass of the vehicle. The longitudinal force is the combination of the braking force, aerodynamic resistance and frictional force, so it should always result in higher values than those obtained using the brake tester, which automatically calculates and subtracts the frictional force. There was no aerodynamic force, as explained earlier in this section.

The closeup look at the maximum braking force zone (Figure 8b) shows a cyclic pattern oscillating between around −9000 N and −14,000 N, due to the ABS activation, with an average longitudinal force of 11,921 N.

The maximum aerodynamic drag during the braking test is calculated as follows:

(29) $$F_{aero.drag}=\frac{1}{2}\cdot C_{d}\cdot A\cdot v^{2}$$

where the following apply:

*C_d_*: Drag coefficient: 0.35 [29].
*A*: Frontal area, (width·height·0.84) [30]: 2.3 m^2^.
*v*: Vehicle speed at maximum *F_x_*: 28 m/s^2^.
*F_aero.drag_* = 316 N

Additionally, the rolling resistance can be calculated as follows:

(30) $$F_{rolling}=C_{r}\cdot m\cdot g$$

where the following apply:

*C_r_*: Rolling coefficient: 0.015 [31].
*m*: Vehicle’s mass at the moment of the test: 1585 kg.
*g*: Acceleration of gravity (9.8 m/s^2^).
*F_rolling_* = 233 N

These results suggest a maximum braking force during the test of 11,372 N.

The brake efficiency calculated with (1) results in

$$E\left(\%\right)=\frac{11,372}{1770\cdot 9.8}\cdot 100=65.59\%$$

This value of brake efficiency results in an 11% increase over the one achieved using the fBrake simulation. Many variables and estimations are involved in these calculations, like the brake tester error, the error linked to the low-cost data acquisition system, and the test track, which, although flat, is not optimal, to name a few. Thus, this 11% difference between the fBrake simulation and the dynamic test can be considered quite a successful result.

### 3.4. Scenario 4: CarSim Simulation

**Figure 10:**
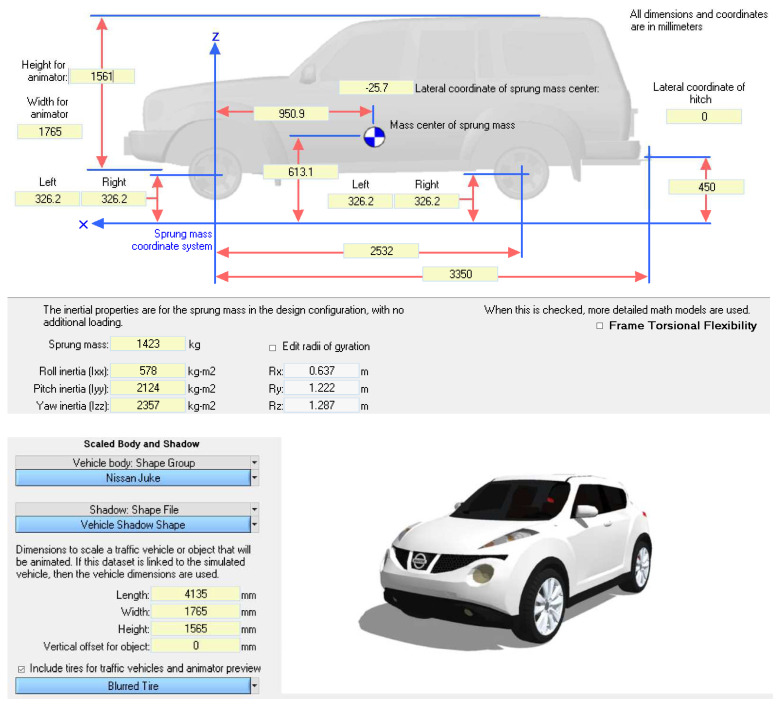
CarSim dimensions and inertial data window, prior to simulation.

For this scenario, inertial data for the specific vehicle needed to be obtained using a vehicle inertia measuring facility. S-E-A Limited selflessly provided the inertial data for the testing vehicle so that this scenario could be evaluated in this research (Figure 10).

**Figure 11:**
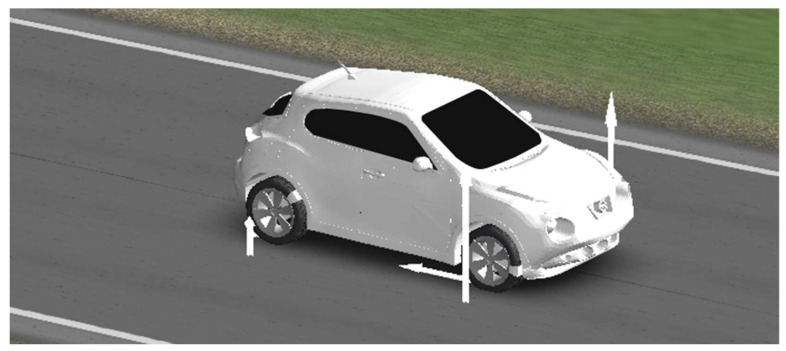
CarSim simulation animation.

With this configuration and these data, longitudinal braking was simulated in a straight line to obtain the individual longitudinal forces for each of the tires. A frame from the simulation animation is shown in Figure 11.

**Figure 12:**
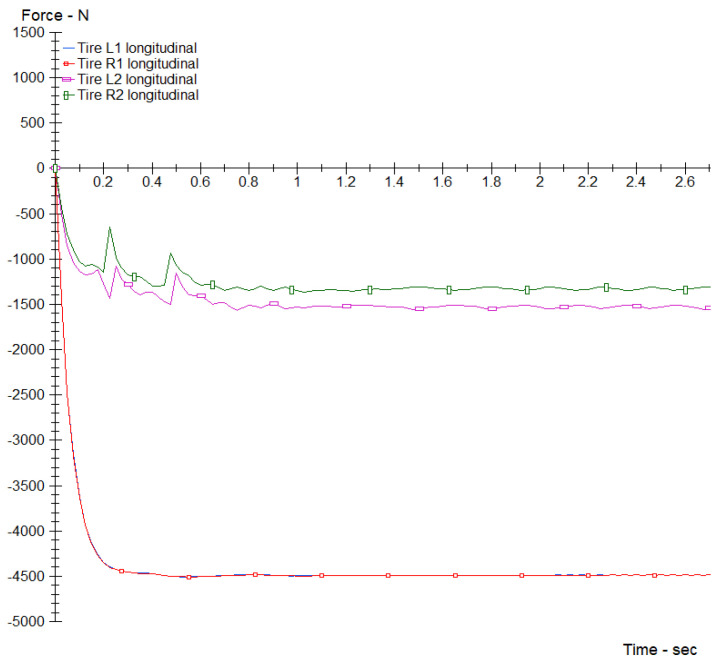
CarSim simulation results: Individual longitudinal braking forces (N) vs. time (s).

The plot of the individual longitudinal brake forces is shown in Figure 12.

The individual maximum brake forces obtained in the simulation are as follows:

Front left tire maximum force: 4480 N.
Front right tire maximum force: 4480 N.
Rear left tire maximum force: 1525 N.
Rear right tire maximum force: 1325 N.
Total maximum braking force: 11,810 N.

The brake efficiency calculated with (1) results in

$$E\left(\%\right)=\frac{11,810}{1770\cdot 9.8}\cdot 100=68.08\%$$

This value of the brake efficiency is close to the result obtained by performing a dynamic brake test. The CarSim results show a 3.8% increase with respect to this result.

### 3.5. CG_h_ and Pitch Moment of Inertia Simulation Results

The results achieved with the simulations performed with the modeled inertial facility in SOLIDWORKS proved an error within 0.11% of the *CG_h_* (Table 5) and 2.2% of the pitch moment of inertia of the vehicle–platform assembly.

#### 3.5.1. *CG_h_* Simulation Results

**Figure 13:**
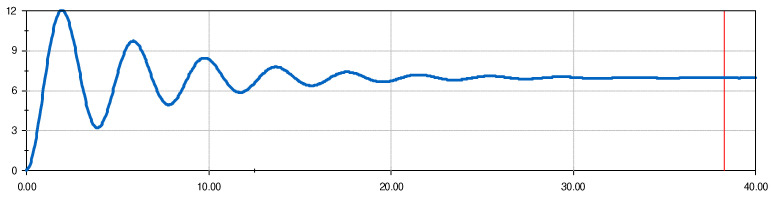
Angle of oscillation (°) over time (s) for the vehicle–platform assembly.

Following the mathematical calculation explained in Section 2.1, the relative angle between the horizontal and the surface of the platform needs to be obtained. Figure 13 shows the process until the equilibrium is eventually achieved, getting a relative angle of 6.87 degrees. The red line shows where the pendulum settles, achieving that angle.

#### 3.5.2. Pitch Moment of Inertia Simulation Results

**Figure 14:**
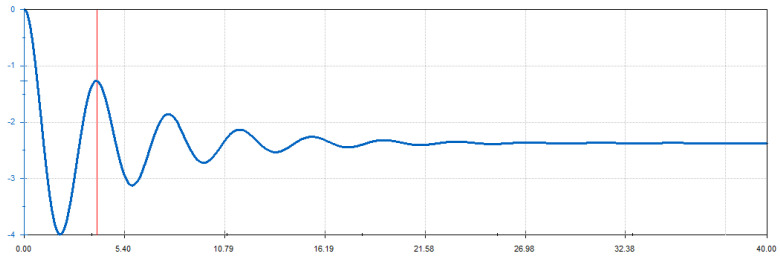
Relative angle of oscillation (°) over time (s) for the vehicle–platform assembly.

For the simulation, the platform was tilted 2.3 degrees and allowed to oscillate. The relative angular displacement between the initial position of the platform and the one it has at each moment in the oscillation is shown in Figure 14. It settled at −2.3 degrees, which implies a return to a completely horizontal equilibrium position.

From the sinusoidal graph, a period T = 3.96 s (marked by the red line) was obtained. The other data are as follows:

*m*: 2966 kg.
*g*: 9.81 m/s^2.^
*r_G_*: 0.554 m.

The moment of inertia with respect to the swing axis can now be obtained using (28):

$$I_{0}=2966\cdot 9.81\cdot 0.554\cdot {\left(\frac{3.82}{2\pi }\right)}^{2}=5958\;kg\cdot m^{2}$$

This moment of inertia is the sum of the vehicle moment of inertia and the platform moment of inertia. To check whether the calculation is correct and the method is valid, it is possible to visualize the moment of inertia of the vehicle–platform pendulum assembly and compare it with that obtained by the pendulum method (Table 6). These MOIs were compared at the CG location of the assembly, so *I*_0_ needed to be displaced using Steiner’s theorem.

$$I_{CG}=I_{0}-m\cdot {r_{G}}^{2}=5958-2966\cdot 0.554^{2}=5048\;kg\cdot m^{2}$$

Similarly, the vehicle-only pitch MOI was obtained (Table 7).

## 4. Discussion

The results of this study show the effectiveness of the fBrake method for calculating the brake efficiency of light vehicles, of both M_1_ (passenger vehicles with nine seats or less, including the driver’s seat) and N_1_ (vehicles for the transport of goods, with a maximum mass not exceeding 3.5 tons) types. This study presents a comparison between four different scenarios in a single vehicle (a partially laden vehicle tested on a brake tester, an unladen vehicle tested on a brake tester, a dynamic brake test on a track using a low-cost data acquisition system and a CarSim simulation using realistic data for the vehicle), but the study also presents analyses far beyond these. After an initial testing plan, including dozens of vehicles from four different segments (sedan, SUV, van and pick-up truck) brought by volunteers to Universidad Carlos III de Madrid facilities and collaborating PTI stations, only a comparison between the first two scenarios was feasible. This paper focuses on the results achieved on a single vehicle in the four different scenarios, even though bootstrapped comparisons between the first two scenarios were performed for every vehicle tested. This latter research will be presented in future papers.

The fBrake method was proven to be a successful method to solve current problems in PTI stations. First, it proved to be effective, since the calculated efficiencies show a small error, but it also proved to be efficient, since it avoids the rejection of vehicles that fulfill the minimum braking efficiency if they are laden, but that appear not to meet the requirements when they are unladen in a driver-only configuration. Other solutions to this problem of vehicle rejection, such as manually loading customers’ vehicles with extra weight or asking them to come back with extra passengers or weight in their cars or vans, would be intrusive and time-consuming.

The results of this research show how a 2015 Nissan Juke would not meet the requirements established by Directive 2014/45/EU [7], according to which a 50% minimum brake efficiency should be achieved. This vehicle, in a driver-only configuration test at a facility, obtained an efficiency of 45.52%, whereas with fBrake, this number increased to 59.68%. It was also demonstrated that two different loading conditions of the vehicle led to similar results, achieving 58.39% in the partially loaded condition, even though brake testers introduce some error to fBrake calculations due to their intrinsic lack of precise repeatability, which is not related to the fBrake methodology and arises each time a new test is performed. Even though load cells are calibrated periodically within tight tolerance levels, the behavior between the wheels and the rollers, as well as that between the wheels and the middle roller that detects the skid, is never the same, and the roller brake tester will end the test slightly sooner or later than the last time, showing different maximum braking forces. Additionally, during the static testing period, for all the vehicles analyzed, it was noticed that some of the brake testers were not able to measure the braking force on the rear axle effectively if the surface on which the front axle was placed was not slippery enough. This may lead to a situation in which the car is not exiting the rollers, not because of the braking force of the rear axle but because of a combination of both axles, leading to a higher value for the braking force on the rear axle than the one it actually has. With fBrake for M_1_ and N_1_ vehicles equipped with EBD, this is solved because the software only needs the data for the front axle and is always on the safety side, since it does not allow a braking force higher than that which the vehicle can provide. The main reason why fBrake for light vehicles is conservative is because of its hypothesis of an invariant rolling resistance coefficient between the partially loaded configuration and the simulated laden one. The changes in the tire diameter and the speed of the wheel are two factors that can vary the rolling resistance coefficient, but they are almost constant between the partially loaded and the laden conditions. Additionally, the tires were the same between the tests, so even though low-rolling-resistance tires may have a lower coefficient, this did not play an important role in the comparative results between the tests. The parameters quantitively affecting the rolling resistance coefficient that changed the most were the weight applied to the wheels and, hence, the tire deformation, especially on the rear axle. Most of the tested vehicles have ABS and EBD. Thus, only the front axle needed to be tested using the roller brake tester with the fBrake method. The vertical load on the front axle varies but, according to our experimentation, the variation is small enough for the rolling resistance coefficient to be considered constant. In fact, the inherent error of the roller brake tester is higher than this difference, inducing a low correlation between them. Different experiments with different loading conditions on the rear axle were performed, and a better correlation was obtained. However, only the front axle needs to be tested in most cases (because of ABS and EBD) and considering the coefficient constant, the results are on the safety side, because the adherence under the laden condition in a testing scenario will always be at least the same as that taken into consideration by the fBrake simulation.

Moreover, the comparison of static testing with dynamic tests and simulations showed similar results, demonstrating that a low-cost data acquisition system using an Apple iPhone XS Max smartphone running MATLAB Mobile, with its IMU running at 100 Hz, is precise enough to achieve comparable results, logging multiple data that are easier to process than using a traditional decelerometer and allowing the user to work with digital data instead of printed graphs and fixed calculations. The CarSim simulations resulted into similar values to those of the dynamic tests, with the former being just 3.8% higher than the latter.

The SOLIDWORKS simulations were used to replicate a realistic facility to obtain the *CG_h_* and pitch moment of inertia. They showed successful results for the method used to calculate both variables, with errors of 0.11% and 5% obtained when simulating the vehicle’s *CG_h_* and the vehicle’s pitch moment of inertia, respectively.

## 5. Conclusions

The findings of this study lead to several key conclusions:

- fBrake was proven to be an effective method of simulation to measure the braking efficiency of an unladen or partially loaded M_1_ or N_1_ vehicle, achieving similar results to a dynamic braking test, but being slightly conservative, achieving a braking efficiency of 59.68%, whereas a dynamic test obtained 65.59% and CarSim simulation obtained 68.08%. This difference is acceptable, since it demonstrates that the vehicles meet the required minimum braking efficiency while remaining on the safety side. However, more advancements will be developed to improve the method and the factors influencing this difference.
- fBrake simulation is easy and safe to operate by Periodic Technical Inspection (PTI) facilities staff and non-intrusive for both customers who attend inspections and their vehicles. This makes the method not only effective, but also efficient.
- The fBrake method requires only front axle measurement for vehicles equipped with EBD. This makes the process not only simpler, but also more consistent, since some brake testers tend to show a higher brake force on the rear axle if the front one is not on a sufficiently slippery surface that facilitates the exit of the rear axle.
- Vehicles without ABS and EBD can also be simulated with fBrake. In these cases, both axles need to be tested on the roller brake tester.
- Dynamic brake tests can be performed effectively using a low-cost data acquisition system with a phone running MATLAB Mobile, a sample rate of 100 Hz and a custom code to plot the results.
- A simulation of the method to estimate the *CG_h_* and the pitch moment of inertia of a vehicle was performed using SOLIDWORKS, achieving errors within 0.11% and 5%, respectively.

## Figures and Tables

**Table 1:** Minimum allowed efficiencies [7].

Date of First Registration Before 1 January 2012		Date of First Registration After 1 January 2012	
N_1_	45%	N_1_	50%
M_1_	50% (^1^)	M_1_	58%

(^1^): 48% for vehicles not fitted with ABS or type-approved before 1 October 1991.

**Table 2:** Front axle braking force for the five initial runs to be replicated (partially laden condition).

Scenario 1 (Laden)					
	Test 1	Test 2	Test 3	Test 4	Test 5
*F_xf_* (N)	5250	5180	5270	5210	5320

**Table 3:** Front axle braking force for the five initial runs to be replicated (unladen condition).

Scenario 2 (Unladen)					
	Test 1	Test 2	Test 3	Test 4	Test 5
*F_xf_* (N)	5030	4960	5110	5050	4990

**Table 4:** 2015 Nissan Juke specifications and PTI conditions for scenarios 1 and 2.

	Scenario 1	Scenario 2
Wheelbase (mm)	2530	
MiRO/MRO (kg)	1370	
*MAM* (kg)	1770	
Front axle *MAM* (kg)	985	
Rear axle *MAM* (kg)	830	
Vehicle height (mm)	1565	
*F_xd_* on inspection (N)	5236	5033
Front weight on inspection (kg)	915	860
Rear weight on inspection (kg)	670	490
Dynamic *F_xf_* at PTI load (N)	6487	6309
Dynamic *F_xr_* at PTI load (N)	2542	2090
fBrake-simulated static *F_xd_* (N)	5321	5498
fBrake-simulated static *F_xd_* (N)	4698	4854
Brake efficiency referred to MRO with PTI-obtained *F_x_* (%)	67.56%	58.81%
Brake efficiency referred to *MAM* with PTI-obtained *F_x_* (%)	52.29%	45.52%
Brake efficiency referred to *MAM* with fBrake-simulated *F_x_* (%)	58.39%	59.68%

**Table 5:** Error calculation between the theoretical *CG_h_* and the simulated one.

Angle	6.87°	0.12 Rad
Calibrated weight position	1965.90 mm	
Swing position	97.63 mm	
Calibrated mass	99.98 kg	
Swing mass	1646.57 kg	
Vehicle mass	1319.42 kg	
Simulated CG → swing pin distance	226.74 mm	
Theoretical CG → swing pin distance	226.48 mm	
Error	−0.11%	

**Table 6:** Theoretical vs. simulated pitch moment of inertia of the vehicle–platform assembly.

Theoretical Pitch MOI (kg·m^2^)	Simulated Pitch MOI (kg·m^2^)	Error (%)
5159	5048	2.2%

**Table 7:** Theoretical vs. simulated pitch moment of inertia of the dummy vehicle.

Theoretical Pitch MOI (kg·m^2^)	Simulated Pitch MOI (kg·m^2^)	Error (%)
2446	2324	5%

## Data Availability

The original contributions presented in the study are included in the article, further inquiries can be directed to the corresponding author.
